# Transmissibility of mpox to the general population from travellers returning to South Korea

**DOI:** 10.1093/jtm/taad080

**Published:** 2023-06-12

**Authors:** Dayeong Lee, Sangbum Choi, Hyunkyung Do, Achangwa Chiara, Min-Kyung Kim, BumSik Chin, Sukhyun Ryu

**Affiliations:** Department of Preventive Medicine, Konyang University College of Medicine, Daejeon 35365, South Korea; Department of Statistics, Korea University, Seoul 02841, South Korea; Department of Preventive Medicine, Konyang University College of Medicine, Daejeon 35365, South Korea; Department of Preventive Medicine, Konyang University College of Medicine, Daejeon 35365, South Korea; Division of Infectious Diseases, Department of Internal Medicine, National Medical Center, Seoul 04564, South Korea; Division of Infectious Diseases, Department of Internal Medicine, National Medical Center, Seoul 04564, South Korea; Department of Preventive Medicine, Konyang University College of Medicine, Daejeon 35365, South Korea

**Keywords:** Monkeypox, public health emergency, MSM, sexually transmitted diseases, travel, spread, transmission

## Abstract

Based on data from three imported mpox cases in South Korea, the overall attack rate was determined to be 1%, while a secondary attack rate of 14% was estimated in the high-exposure group.

In East Asia, mpox was initially detected in June 2022 (South Korea) among returning travellers who had engaged in sexual activity with men from Europe. As of May 2023, several European countries had reported a single-digit incidence of mpox infections, seemingly indicating the end of the epidemic.^1^ By contrast, East Asia, including China, Japan, South Korea and Taiwan, has continued to report an increase in the incidence of mpox infections.^1^

A recent study demonstrated the importance of prioritizing public health and social measures, including mpox vaccination of high-risk subpopulation, to reduce mpox transmission across the community.^2^ However, epidemiological data on Asian countries remain lacking.^3^^,^^4^ Therefore, this study examined the transmissibility (i.e. attack rate) of mpox using contact-tracing data from the first three imported cases in South Korea.

We collected publicly available data on mpox cases from Korean public health authorities (Supplementary Material). Based on the contact-tracing data of the imported mpox cases, the degree of exposure among individuals who had come into contact with the confirmed cases was categorized as follows: ‘high degree’, ‘intermediate degree’ and ‘low degree’ (Supplementary Material).^5^

We measured the overall attack rate, which is the probability that an infection occurs among susceptible individuals, and the secondary attack rate in the high-exposure group and estimated the 95% confidence intervals (CIs) using the bootstrap method (Supplementary Material).

The overall attack rate based on the first three imported cases was found to be 0.9% (1 out of 106; 95% CI, 0.0–2.8%). After stratification by degree of exposure, the secondary attack rate was estimated at 14.2% among contacts in the high-exposure group (1 out of 7; 95% CI, 0.0–42.9%). However, no transmission cases were identified in the intermediate- and low-exposure groups (Table 1).

**Table 1:** Descriptive summary of the demographic and epidemiological characteristics of the first three imported mpox cases in South Korea

ID	Age, years	Sex	Travel history	Date of symptom onset	Date of entry into Korea	Date of isolation	Overall no. of contacts, *n*	No. of contacts by degree of exposure, *n*		
								High	Intermediate	Low
1	34	Male	Germany	18 June 2022	20 June 2022	21 June 2022	49	0	8	41
2	35	Male	Germany	28 August 2022	18 August 2022	1 September 2022	15	0	2	13
3	24	Female	Dubai	8 November 2022	4 November 2022	15 November 2022	42	7	9	26

Note: Based on the degree of mpox exposure, individuals were categorized into ‘high degree’ (unprotected direct contact with a confirmed case or high-risk environmental contact), ‘intermediate degree’ (unprotected exposure to infectious materials and droplets or potential exposure to aerosols) and ‘low degree’ (protected from physical or droplet exposure or no physical contact and minimal chance of exposure to droplets) groups.^5^

The secondary attack rate provides useful information on how the interactions in a specific setting relate to the transmission risk. Our findings suggest that mpox transmissibility from the first three imported cases to the South Korean population was low and that mpox transmission was potentially driven by the high-exposure group. This finding is consistent with those of previous studies in that the major risk for mpox transmission was associated with a high degree of exposure, including sexual contact.^4^ Therefore, to prevent delayed diagnoses and reporting, mpox vaccination and awareness campaigns among high-risk populations need to be prioritized.^6^

Notwithstanding, this study has certain limitations. First, behavioural heterogeneity might have influenced the estimation of the attack rate. Thus, further research involving a greater number of cases with contact-tracing data is warranted to avoid possible bias and to reduce CI width. Second, a secondary mpox infection from the imported case occurred during medical practice.^7^ Therefore, the secondary attack rate in our study might have been overestimated.

The continual estimation of mpox transmissibility is important for evaluating control measures against mpox. A comparison of transmissibility estimates according to different mpox-exposure levels and modes of transmission (e.g. homosexual or bisexual contact) is warranted. Furthermore, with the easing of SARS-CoV-2-related international travel measures in Asian countries,^8^ travel advice and active case findings of mpox for the travellers returning from mpox-affected countries are necessary to limit the mpox importation risk.

## Funding

This work was supported by the Basic Science Research Program of the National Research Foundation of Korea of the Ministry of Education (NRF-2020R1I1A3066471).

## Authors’ contributions

S.R. conceived the study. D.L., H.D., A.C., M.-K.K. and B.C. were involved in data collection and assimilation. D.L., S.C., H.D., A.C. and S.R. performed data analysis, discussed the results and drafted the manuscript. All authors have critically read and approved the final manuscript.

## CRediT author Statement

Dayeong Lee (Data curation [equal], Formal analysis [equal], Project administration [equal], Writing—original draft [equal]), Sangbum Choi (Formal analysis [equal], Methodology [equal], Writing—review & editing [equal]), Hyunkyung Do (Data curation [equal], Formal analysis [equal], Writing—original draft [equal]), Achangwa Chiara (Validation [equal], Writing—review & editing [equal]), Min-Kyung Kim (Validation [equal], Writing—review & editing [equal]), BumSik Chin (Validation [equal], Writing—review & editing [equal]), and Sukhyun Ryu (Conceptualization [lead], Formal analysis [lead], Project administration [lead], Supervision [lead], Writing—original draft [lead], Writing—review & editing [equal])

**Conflict of interest**: S.R. was supported by the Korea Disease Control and Prevention Agency (contract numbers: 2023020748C-00 and 2023040522C-00). All other authors report no potential conflicts of interest.

## Ethical approval and consent to participate

This study did not require institutional review board approval or informed consent because all data used are anonymous and are publicly available on websites.

## Data availability

The data underlying this article will be shared on reasonable request to the corresponding author.

## Supplementary Material

Supplementary_Materials_taad080
