# Supplementary material for: Transmissibility of mpox to the general population from travellers returning to South Korea
Source: J Travel Med. 2023 Jun 12;30(5):taad080. doi: 10.1093/jtm/taad080 (PMC10481410; doi:10.1093/jtm/taad080)
Supplement: Supplementary_Materials_taad080 [file supplementary_materials_taad080.docx]

Supplementary

**Transmissibility of mpox to the general population from travellers returning to South Korea**

# Definition of mpox case in South Korea

After experiencing a vast Middle East respiratory syndrome coronavirus outbreak initiated by a traveller returning from the Middle East in 2015, South Korea instated a national surveillance program involving virological mpox testing to rapidly detect mpox-infected inbound travellers. A suspected mpox case was defined as an individual who presented with a vesicular or pustular rash on their skin with an epidemiological link to a recently visited mpox-affected country or sexual contact with a confirmed case.^1^ A confirmed mpox case was defined as a person with laboratory-detected mpox infection.^1^

# Definition of exposure level

Based on the contact history of the confirmed cases, the degree of exposure among individuals who had come into contact with the confirmed cases was categorized as follows: ‘high degree’ (unprotected direct contact with a confirmed case or high-risk environmental contact), ‘intermediate degree’ (unprotected exposure to infectious materials and droplets or potential exposure to aerosols), and ‘low degree’ (protected from physical or droplet exposure or no physical contact and minimal chance of exposure to droplets).^2^

# Bootstrap method

Bootstrapping is a resampling method that uses data from one sample to generate a sampling distribution by repeatedly obtaining random samples from the known sample, with replacement.^3^ We generated 1000 bootstrap samples obtained from the original data with the same sample size and computed the mean of each bootstrap sample. Thereafter, we determined the 95% confidence interval for the mean of each original data from the 2.5^th^ and 97.5^th^ percentiles of the mean bootstrap sample estimates. Analyses were done in R version 4.1.3 (R Foundation for Statistical Computing, Vienna, Austria).

# References

1 Korea Disease Contral and Prevention Agency. Mpox. Korea Disease Contral and Prevention Agency, 2023. https://www.kdca.go.kr/contents.es?mid=a20108060000 (5 June 2023, date last accessed).

2 Centers for Disease Control and Prevention. Monitoring and risk assessment for persons exposed in the community. US Centers for Disease Control and Prevention, 2023. https://www.cdc.gov/poxvirus/mpox/clinicians/monitoring.html (5 June 2023, date last accessed).

3 JM Bland, DG Altman. Statistics notes: Bootstrap resampling methods. BMJ 2015; 350:h2622. https://doi.org/10.1136/bmj.h2622
